# Pancreatic tuberculosis mimicking pancreatic malignancy in a young male: a rare case report

**DOI:** 10.1186/s12879-026-13399-z

**Published:** 2026-06-19

**Authors:** Kiran Shahzadi, Ahmad Raza, Nasim Akhtar, Amina Saddiqa, Arooj Sikandar Malik, Umair Ajmal

**Affiliations:** Department of Infectious Diseases, Pakistan Institute of Medical Sciences, Islamabad, Pakistan

**Keywords:** Pancreatic tuberculosis, Pancreatic mass, Pancreatic malignancy, Extrapulmonary tuberculosis, Endoscopic ultrasound (EUS), Fine-needle aspiration biopsy, GeneXpert MTB/RIF

## Abstract

**Background:**

Isolated pancreatic tuberculosis is a rare form of extrapulmonary TB, often misdiagnosed as malignancy due to overlapping clinical and radiological features.

**Case presentation:**

We report a case of a 24-year-old male presenting with right hypochondrial pain, jaundice and weight loss. A mass was found in the head of pancreas raising strong suspicion for pancreatic cancer. Endoscopic ultrasound guided biopsy was done and histopathology of the tissue revealed caseating granulomas, and GeneXpert revealed Mycobacterium tuberculosis. The patient responded well to anti-tuberculous therapy, with clinical and radiological improvement.

**Conclusion:**

This case underscores the importance of considering tuberculosis in the differential diagnosis of pancreatic masses, especially in endemic regions.

**Clinical trial number:**

Not applicable.

## Introduction

Tuberculosis (TB) can manifest as pulmonary or extrapulmonary disease. It remains a significant global health concern, with the World Health Organisation (WHO) estimating 10.8 million cases worldwide in 2023, predominantly in low- and middle-income countries [1]. One of the rare extrapulmonary manifestations of TB is pancreatic TB, which is reported to be present in 0.2 to 2% of all cases [2]. Isolated pancreatic TB, however, is exceptionally uncommon [3]. Reasons for its rarity include the pancreas’s retroperitoneal location and presence of pancreatic enzymes that inhibit mycobacterial growth [3].

Diagnosis of pancreatic TB can be established through imaging-guided biopsy with histopathology and molecular testing. However, it may clinically and radiographically mimic pancreatic malignancy [4], leading to diagnostic dilemmas and unnecessary surgical interventions. In many cases, TB has been discovered only by histopathological and molecular testing post exploratory laparotomy for a suspected pancreatic cancer [5]. Here we present a case of a 24-year-old man who was initially suspected to have a pancreatic neoplasm but was found to have pancreatic TB after his diagnostic work-up.

## Case presentation

A 24-year-old unmarried Pakistani male with no significant past medical history presented with a 5–6-month history of right hypochondriac pain, progressive jaundice, and a 5 kg weight loss. He denied fever, cough, night sweats, anorexia, pruritus, or bleeding tendencies. He was not taking any regular medication. The patient reported no history of alcohol or illicit drug use. Family history was notable for tuberculosis. Physical examination revealed deep icterus. Abdomen was soft and non tender; no palpable mass or visceromegaly was appreciated.

His baseline investigation showed haemoglobin of 14.7 g/dl with the total leucocyte count of 6510/uL and platelet count of 216,000/uL, total bilirubin 9.53 mg/dl, direct bilirubin 8.40 mg/dL, Alanine aminotransferase (ALT) 570 U/L, Aspartate aminotransferase(AST) 354 U/L, alkaline phosphatase(ALP) 976 U/L, amylase 76.8 U/L, urea 16.4 mg/dl, creatinine 1.04 mg/dl, sodium 135mEq/L, potassium 4.65mEq/L.HbsAg, AntiHCV, and HIV antigen/antibody combo were negative.

**Fig. 1:**
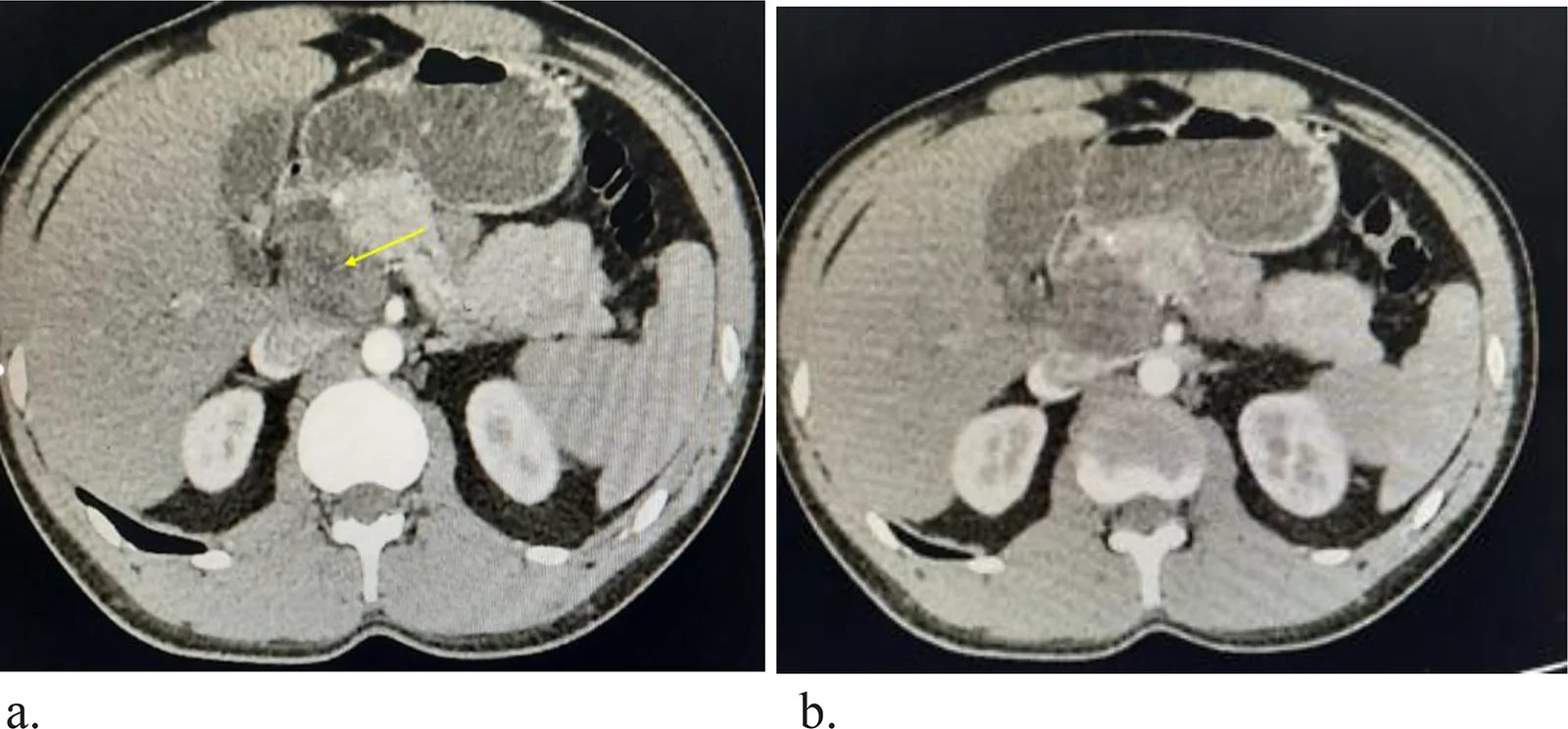
(**a**, **b**) CT scan with contrast of the abdomen.Arrow in the Fig 1a points to a heterogeneously enhancing hypodense lobulated mass in the uncinate process and the head of the pancreas

Chest X-ray was unremarkable. Abdominal ultrasound revealed a 26 × 29 × 33 mm mass in the pancreatic head. Contrast-enhanced CT of the abdomen showed a well-circumscribed, heterogeneously enhancing hypodense lobulated mass involving the uncinate process, head of the pancreas, and caudate lobe of the liver, causing complete encasement of the mid-common bile duct with mild intrahepatic biliary dilation as shown in Fig. 1 (a, b) below. There was more than 90% encasement of the right hepatic artery and close abutment of adjacent structures, including the main portal vein, common hepatic artery, inferior vena cava, and superior mesenteric artery. These findings raised suspicion of a neoplastic process.

Endoscopic ultrasound (EUS) identified a 3 cm hard peripancreatic necrotic mass. Tissue biopsy was performed using a 22G needle, and samples were sent for histopathology and Mycobacterium tuberculosis polymerase chain reaction (PCR). Endoscopic retrograde cholangiopancreatography (ERCP) was subsequently performed to relieve biliary obstruction, and cannulation of common bile duct was attempted which was unsuccessful. A temporary 5 Fr × 7 cm stent was placed in the pancreatic duct to prevent post-ERCP pancreatitis, which was removed two weeks later.

**Fig. 2:**
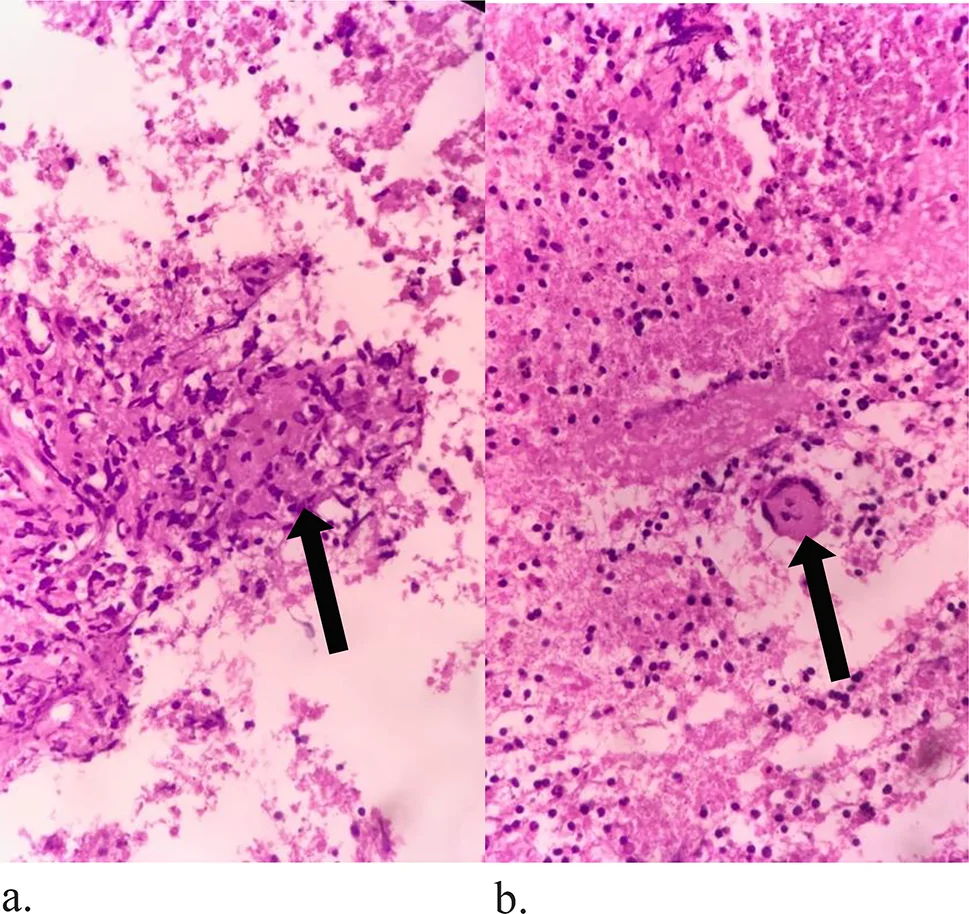
(**a**, **b**): Histopathology slides showed extensive caseating necrosis with well-formed epithelioid granulomas. Extensive caseating necrosis with well-formed epithelioid granulomas surrounded by dense inflammation comprising neutrophils, lymphocytes, and giant cells which are shown in Fig. 2 (**a**, **b**)

Histopathological examination revealed extensive caseating necrosis and well-formed epithelioid granulomas (Fig. 2). Ziehl-Neelsen staining for acid-fast bacilli and periodic acid–Schiff and Grocott’s methenamine silver stains for fungal elements were negative.

No evidence of malignancy was found. Gene Xpert testing on peripancreatic tissue detected Mycobacterium tuberculosis DNA without rifampicin resistance, confirming the diagnosis of pancreatic tuberculosis. The patient was initiated on antituberculous therapy (ATT consisting of oral isoniazid (5 mg/kg/day), rifampicin(10 mg/kg/day), ethambutol (15 mg/kg/day), and pyrazinamide (20/kg/day) for the initial two months, followed by isoniazid and rifampicin for the subsequent four months. He attended regular follow-up visits. The patient demonstrated marked improvement in his pain and jaundice in the following months. The improving trend of his liver function tests is depicted in Table 1.

**Table 1:** Trend of liver function tests post the start of treatment

Bilirubun in mg/dL (normal 0.2–1.1)	Before Intervention	2 weeks after the start of antitubercular therapy	After 2 months (end of intensive phase)	After 6 months (end of antitubercular therapy)
	9.53	1.41	0.872	0.40
Alanine transaminase in U/L (normal 5–45)	570	253	76.2	38.7
Aspartate transaminase in U/L (normal 8–33)	354	181	61.3	30.1
Alkaline phosphatase in U/L (normal 50–150)	976	613	264	126

**Fig. 3:**
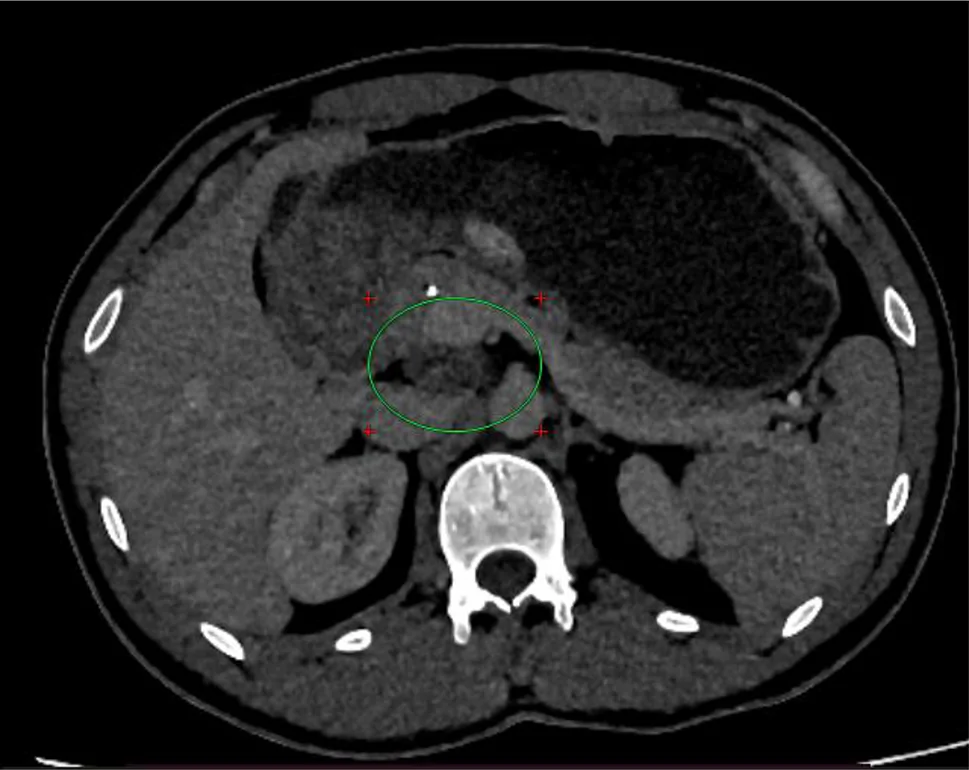
Contrast enhanced CT scan of the abdomen showed significant reduction of the lesion

A repeat contrast-enhanced CT abdomen after six months of therapy demonstrated a significant reduction in the size of the pancreatic mass, as depicted in Fig. 3.

## Discussion

Pancreatic TB should be an important differential to consider along with neoplasm in patients presenting with weight loss, obstructive jaundice and biochemical and imaging evidence of pancreatic mass, especially in endemic areas. Pancreatic TB occurs in a wide demographic. Most cases occur in immunocompetent adults, and patients are more commonly male [5, 6], a group to which our patient belonged. The most common symptoms are epigastric pain, weight loss, anorexia, vomiting, fever, jaundice, night sweats and fatigue [5]. Symptoms can vary, like in the case of our patient, who did not report any anorexia, vomiting, fever, night sweats or fatigue. Varying presentations add to the diagnostic complexities; clinicians in endemic areas should always remain mindful of the probability of pancreatic TB.

Our patient’s mass involved the head of the pancreas and surrounding tissues, which is the most common site according to literature [5]. Due to overlapping clinical and radiological features with malignancy, making sound management plans can be challenging, especially considering the urgency associated with potential malignancy [7]. This has led to cases where surgical interventions for potential malignancy, like exploratory laparotomy, were performed and TB was discovered only afterwards on tissue histopathological and molecular analysis [8–10]. Careful evaluation, along with remaining conscious about possible TB, can help clinicians in managing the condition better while avoiding any unwanted invasive treatment.

We were able to diagnose pancreatic TB through analysis after EUS-guided tissue biopsy. The patient was placed on 2 months of rifampicin, isoniazid, pyrazinamide, and ethambutol, followed by 4 months of rifampicin and isoniazid (2HRZE/4RH). At the end of 6 months, the patient’s mass saw almost complete resolution on contrast-enhanced CT scan. Similar approaches have been documented previously. For example, a 32-year-old female patient with pancreatic TB in the head of the pancreas was treated with 2HRZE/7RH, and showed significant improvement in symptoms and complete resolution of mass on CT scan [8]. A 26-year-old man treated with 2HRZE/7RH showed clinical improvement in 2 weeks and complete resolution of pancreatic lesion with residual fibrosis in a six-month CT scan [2]. Another 46-year-old patient diagnosed similarly showed excellent response to antitubercular therapy [11].

Our case shows that clinicians in TB-endemic regions should have a high index of suspicion for pancreatic TB. It can help avoid unnecessary interventions, save precious resources, and lead to better management and patient outcomes.

## Limitations

This report describes a single case from one medical center, so the findings cannot be applied to all patients. Our follow-up focused on the patient’s early clinical and radiological response, and we did not evaluate longer-term outcomes. Although Mycobacterium tuberculosis was confirmed through GeneXpert and caseating granulomas were seen on histology, other microbiological tests such as acid-fast staining may have lacked sensitivity in this setting, leading to negative smear results. These factors should be kept in mind when interpreting the diagnostic process and the patient’s outcome.

## Conclusion

This case highlights the importance of considering pancreatic tuberculosis in the differential diagnosis of pancreatic masses in young patients, especially in TB-endemic regions. Accurate diagnosis through histopathological and molecular testing allows for prompt initiation of ATT, avoiding unwarranted surgical procedures. Early recognition and appropriate treatment lead to excellent outcomes, as demonstrated in our patient. 

## Data Availability

No datasets were generated or analysed during the current study.
